# Comparing alternative methods of measuring cumulative risk based on multiple risk indicators: Are there differential effects on children’s externalizing problems?

**DOI:** 10.1371/journal.pone.0219134

**Published:** 2019-07-03

**Authors:** Idean Ettekal, Rina D. Eiden, Amanda B. Nickerson, Pamela Schuetze

**Affiliations:** 1 Department of Educational Psychology, Texas A&M University, College Station, TX, United States of America; 2 Clinical and Research Institute on Addictions, University at Buffalo, the State University of New York, Buffalo, NY, United States of America; 3 Department of Psychology, University at Buffalo, State University of New York, Buffalo, NY, United States of America; 4 Alberti Center for Bullying Abuse Prevention, University at Buffalo, the State University of New York, Buffalo, NY, United States of America; 5 Department of Psychology, State University of New York Buffalo State, Buffalo, NY, United States of America; Monash University, AUSTRALIA

## Abstract

This study examined several alternative methods to measure cumulative risk (CR) based on multiple risk indicators. Several methods for measuring CR are presented and their conceptual and methodological assumptions are assessed. More specifically, at the individual risk level, we examined the implications of various measurement approaches (i.e., dichotomous, proportion- and z-scores). At the composite level, we measured CR as an observed score, and compared this approach with two variable-centered approaches (consisting of reflective and formative indicators) and two person-centered approaches (consisting of latent class analysis and latent profile analysis). A decision tree was proposed to aid researchers in comparing and choosing the alternative methods. Using a sample of 169 low-income families (children approximately 5 years old, 51% girls; 74% African American, and their primary caregiver), we specified models to represent each of the alternative methods. Across models, the multiple risk composite was based on a set of 12 individual risk indicators including low maternal education, hunger, meal and money unpredictability, maternal psychopathology, maternal substance use, harsh parenting, family stress, and family violence. For each model, we estimated the effect size of the composite CR variable on children’s externalizing problems. Results indicated that the variable-centered CR composites had larger effects than the observed summary score CR indices and the person-centered methods.

## Introduction

Investigating the risk factors associated with children’s psychological adjustment has been an ubiquitous aim in the psychological and developmental sciences. Related to this aim, one line of investigation has focused on how children exposed to multiple co-occurring risk factors tend to have worse developmental outcomes than those who have less, or no, risk factor exposure. Rutter [1,2] proposed a method for measuring *cumulative risk (CR)* in which multiple risk factors are aggregated to create a single composite index of risk. This method has been widely used by researchers and is a robust predictor of children’s mental health problems and psychological adjustment (for a review, see Evans, Li, & Whipple, 2013[3]).

Researchers have articulated several conceptual and methodological arguments for aggregating multiple risk indicators into a single composite, or CR, variable. Conceptually, aggregating multiple risk indicators can more accurately ascertain how children’s risk exposure may cross multiple systems and domains including parental, familial, home, school and neighborhood contexts [3,4]. For many populations (e.g., children living in low-income, urban contexts), risk exposure across these domains tend to co-occur and rarely exist in isolation [5]. Methodologically, this approach provides a more simplified model than estimating each risk factor as a separate predictor. This may be particularly advantageous when investigators are using small samples sizes, examining exposure to a large number of risk factors within one model, or when some risk factors co-occur and correlate with each other, which may result in concerns about collinearity or suppression effects [6]. Furthermore, the use of an aggregate score typically has greater predictive power than any single risk factor [3]. However, when this is not the case, it would justify using a single risk factor as opposed to aggregating multiple risk indices. Investigators have also reported threshold effects, which are not apparent when examining individual risk factors [7]. Threshold effects indicate that the effect of multiple risk indicators is exacerbated when the number of risks to which a child is exposed exceeds a given threshold, thus CR exposure may have a non-linear association with children’s psychological adjustment.

Since the inception of the CR approach, there have been numerous methodological advances that can be readily applied to measure children’s exposure to multiple risk factors. However, comparisons across varying methods have been rare and most studies have typically relied on one method of measuring CR. The aims of this study were to provide a conceptual and methodological comparison of several alternative methods that can be applied for measuring multiple risk exposure with the goal of assisting researchers in considering the nuances and distinctions of various approaches. From a conceptual standpoint, several comparisons are made across each method that is evaluated. More specifically, we consider, 1) the assumptions of each approach with respect to whether individual risk indicators should be measured along a continuum or dichotomized to differentiate those with the most severe risk, and 2) the implications of each approach with respect to the relative influence (weight) of individual risk indicators in forming a composite variable. From a methodological standpoint, we present a strategy for applying each method and elaborate on the conceptual assumptions that underlie each method. Since one of the primary aims of studies which incorporate a multiple risk approach is to predict a specific outcome of interest, we evaluate the predictive utility of each method on children’s externalizing problems in a low-income, diverse sample at high risk due to maternal substance use during pregnancy. Because there are numerous co-occurring risks that are present in this population, this was an ideal sample to examine the various methods of assessing children’s multiple risk exposure.

**Fig 1:**
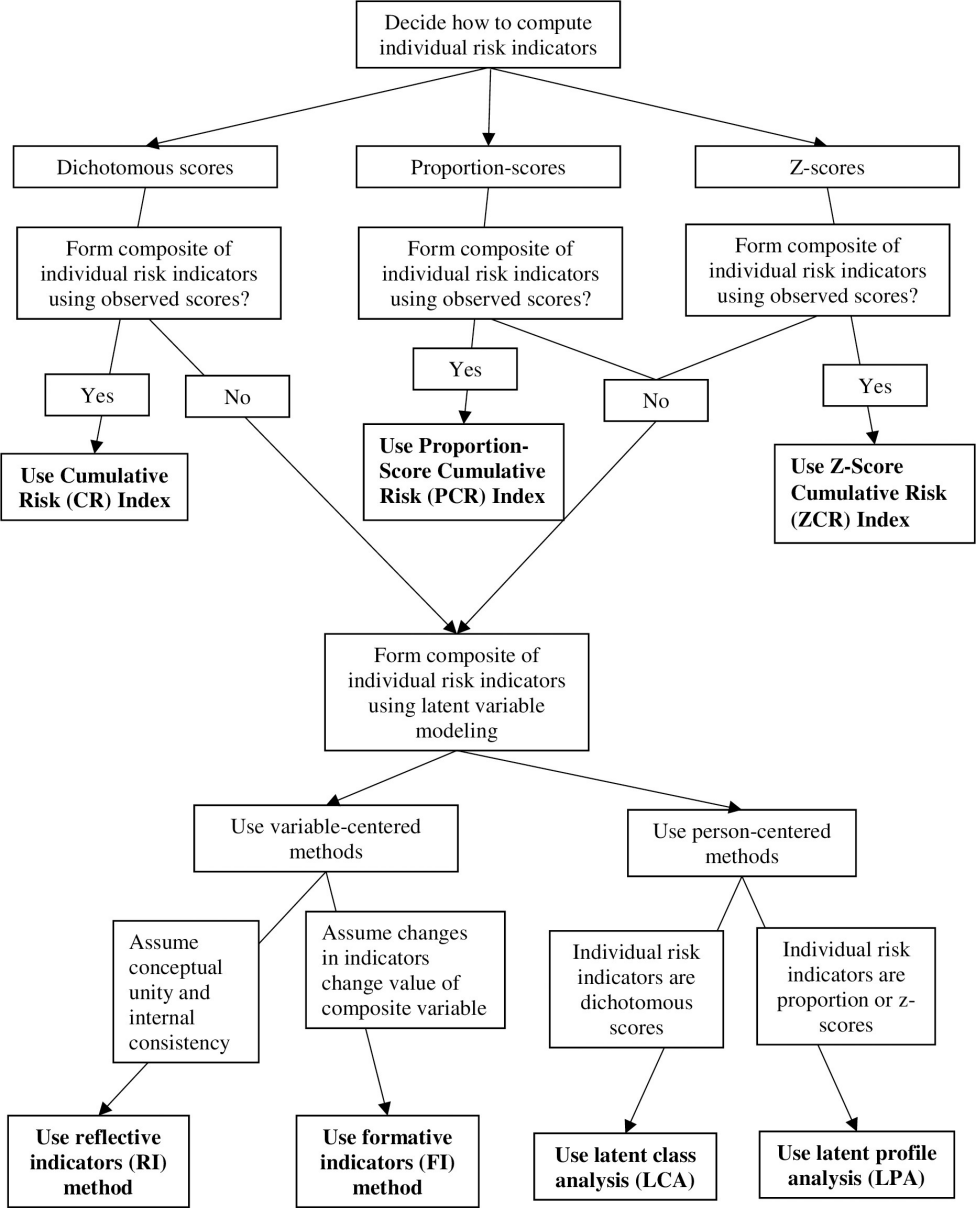
Decision tree illustrating alternative methods to measure cumulative risk.

It should be emphasized that the goal of this study was not to recommend a single approach, or propose a ‘gold standard’ for measuring CR, but rather to highlight various methods that have been used by investigators. By evaluating and comparing the conceptual and methodological assumptions, and potential strengths and limitations of these methods, this study aims to assist researchers in becoming more knowledgeable about the various options that are available for assessing CR, and better equipped to make a more informed decision regarding their choice of method. Depending on how researchers choose to conceptualize and measure CR, and their specific research objectives (e.g., analytic and research designs), there are likely instances in which each of these methods can be effectively and appropriately applied. Accordingly, we propose a decision-tree that can facilitate this process (see Fig 1).

### Observed score methods

#### Cumulative risk (CR) index

The conventional cumulative risk (CR) index typically relies on a set of dichotomous or binary variables, each of which is coded to reflect the presence or absence of a particular risk factor (i.e., *1 = high risk*, *0 = no or low risk*). This index is computed by summing each of the risk indicators such that higher scores reflect greater exposure to multiple risk factors. This summed composite variable (referred to here as the CR index) can then be incorporated into other analyses (e.g., regression models, structural equation modeling) as a predictor variable.

At the individual risk level, the investigator must take several factors into consideration. Decisions should be made a priori about how each variable should be dichotomized. For some variables (e.g., categorical data), the variable may already be measured at the binary level. For example, low maternal education may be measured by the presence or absence of a high school diploma, or single parenthood may be measured by the presence or absence of a second caretaker residing in the child’s home. However, in many instances, this may require a conversion of a continuous scale variable into a dichotomous variable. In these instances, investigators should have a rationale for determining a cut-off score (e.g., a median split, upper quartile, threshold score) or criterion by which to dichotomize a continuous variable. Notably, dichotomization of continuous variables carries certain drawbacks which investigators should take into consideration [8, 9]. Because this approach uses dichotomized individual risk indicators, it does not account for individual risk indicators that may span a continuum. Stated differently, this approach tends to measure the highest or most severe exposure to a risk factor as opposed to differentiating a risk factor along a continuum that differentiates severity. For instance, using an upper quartile split (i.e., 75^th^ percentile and above are considered to be ‘at risk’), it is assumed that those who fall in the 50^th^ to 75^th^ percentile have ‘no risk’ similar to those who fall in the 0 to 25^th^ percentile range. Depending on how an investigator dichotomizes the individual risk indicators, this approach may be sample-specific. For instance, the use of an upper quartile split may constitute very different risk classifications in a high-risk or low-risk sample.

At the composite level, this approach also assumes that when risk is present, each individual risk variable carries the same weight toward the aggregated CR index. That is, this approach does not differentially weigh individual risk factors such that some indicators may have a stronger influence on the composite than others. Conceptually, the CR index is based on the premise that risk exposure becomes more detrimental based on the *number* of different risks a child experiences, rather than the specific *form* or *nature* of risk. Thus, imposing equal weights appears consistent with this premise.

One of the potential advantages of the CR index is that it is easily interpretable. To illustrate, supposing the unstandardized coefficient of the CR index is equal to .2, this can be easily interpreted to suggest that for a one-unit increase in cumulative risk (which would correspond to the presence of an additional risk), there is a .2 increase in the outcome variable. To the extent there is a threshold effect present, another potential advantage of the CR index is that it can readily detect such effects (e.g., determining the number of risk factors needed for a child to be at greater risk for certain developmental outcomes).

#### Proportion-score cumulative risk (PCR) index

As an alternative to the CR index, another strategy that can be applied is the *proportion-score cumulative risk (PCR) index* [10]. For each risk indicator, a proportion-score is computed by dividing each individual score by the maximum score, yielding a proportion-score with a maximum value of one. At the composite level, the PCR index consists of computing the mean (or sum) of each proportion-score to estimate a composite variable. Compared to the CR index, there may be conditions in which this approach is more or less advantageous. When the individual risk indicators are derived from continuous scale variables, it may be more feasible to transform them into a proportion-score than to dichotomize. Notably, when an individual risk indicator is dichotomous by design, it does not require a transformation to a proportion-score. One of the distinctions of this approach is that it maintains the relative rank-ordering of individuals on a variable which is lost in dichotomization. Thus, unlike the CR index which tends to differentiate the presence of high-risk, this approach assumes risk occurs on a continuum with varying degrees of severity. Because a proportion-score has a maximum possible value of one, this approach is similar to the CR index in that high levels of risk across different risk indicators carry the same maximum weight. Considering that proportion-scores are derived from the maximum score within a particular sample, one of the potential drawbacks of this approach, perhaps more so than the CR index, is that it is sample-specific.

Compared to the CR index, the PCR index has been used infrequently. An exception is a study by Moran et al. (2016), which applied this approach to form a PCR index consisting of low maternal education, single parent status, residential instability, household density, negative life events, parental divorce, and maternal depression. However, these investigators did not explicitly assess the potential advantages of this approach compared to the CR index.

#### Standardized (z-score) cumulative risk (ZCR) index

The standardized (i.e., z-score) cumulative risk index (ZCR) is another approach that shares several similarities with the PCR index (note that this approach is referred to as a *summary score* in Evans et al., 2013). This approach entails transforming each individual risk variable into a standardized score (i.e., z-score), and aggregating the standardized scores (i.e., computing a mean or sum score). It is a feasible approach when the risk indicators are based on continuous scales, and maintains the relative rank-ordering of individuals. Thus, this approach assumes risk occurs on a continuum with varying degrees of severity or risk. Because z-scores are derived from the standard deviation within a given sample, this approach is also highly sample-specific, similar to the PCR.

Unlike the CR or PCR indices, this approach does not inherently assume that all risk indicators are equally weighed. That is, whereas the CR and PCR indices have an imposed range for each individual risk indicator (*0 = no risk*, *1 = high risk*), this is not the case with z-scores. Thus, there may be instances in which certain risk variables (i.e., those in which there is a greater range in standardized scores) contribute more to the composite index. Differential weights may be advantageous in instances in which a researcher presumes that severe exposure on a specific risk indicator (or multiple indicators) may be particularly detrimental for a given outcome, and thus, has a rationale for not assuming equal weights of the risk indicators.

Few investigators have explicitly compared the ZCR to the CR index or other methods. For instance, a systematic review by Evans and colleagues (2013) found only one study which explicitly compared these two methods. This study reported that the ZCR was a stronger predictor than the CR index [11]. However, this finding may be specific to the risk indicators, or outcomes, that were assessed. Thus, there remains a need for additional research to compare these methods.

### Variable-centered methods

Since the inception of the CR index, the application of latent variable models (e.g., structural equation modeling, mixture modeling) in psychological research has expanded considerably allowing investigators to utilize these methods to operationalize multiple risk indicators using latent (*unobserved*) constructs. As illustrated in Fig 1, when investigators decide to forgo the use of observed score CR indices (i.e., CR, PCR and ZCR), they have the option to use latent variable methods. We discuss several variants of models that can be applied to measure multiple risk indicators, including variable-centered methods (i.e., reflective and formative indicators) and person-centered methods (latent class analysis and latent profile analysis). These methods can be applied with dichotomous, proportion- and z-score indicators. Notably, because the proportion and z-score indicators consist of transformations that maintain the same rank-ordering of individuals, they produce equivalent covariance (correlation) matrices. Thus, the differences between these two approaches are perhaps more evident when using observed score CR indices, and less relevant for the latent variable approaches discussed in the following sections which rely on a covariance matrix.

#### Reflective indicator (RI) method

Reflective indicator (RI) models entail specifying a measurement model using multiple risk indicators as *effect indicators* of an unobservable latent construct [12]. This can be achieved by specifying a latent factor such that each individual risk variable is treated as an indicator of the latent multiple risk construct. One of the advantages of this approach is that it can be applied when the individual risk indicators are dichotomous or continuous (i.e., proportion or z-score) variables. Comparing this method to other approaches raises several other considerations. The RI method assumes conceptual unity and that the indicators are inter-correlated, implying that each indicator is reflective of an underlying unobserved latent construct [12]. Depending on how the multiple risk measure is conceptualized and what domain(s) of risk it is intended to measure, this assumption may, or may not, be met. From our viewpoint, the RI method may be problematic when investigators are attempting to measure a single composite variable that is based on conceptually distinct risk processes, which are not necessarily correlated with each other. In these instances, an indicator that is uncorrelated with other indicators may have a diminished effect on the latent construct [13].

The RI method can be conceived as a factor-analytic model. By extension, this model can also be used to specify multiple latent variables (factors) to assess multiple domains of risk. To simplify the comparison of approaches in the current study, we consider the simplest form of this model in which one latent variable (domain) is specified. However, this approach may be particularly advantageous in scenarios in which researchers seek to aggregate the individual risk indicators into multiple, distinct domains [14].

#### Formative Indicator (FI) method

A second variable-centered approach consists of the *formative indicator (FI)* method. The FI method consists of specifying a multiple risk composite variable in which the indicators are predictors of the composite [15], as opposed to being reflective of it (as in the RI approach). The use of formative indicators within psychological research has been a contentious one [13, 16], however, there may be strengths to using this approach to investigate multiple risk processes. Moreover, although this approach has been used infrequently to assess multiple risk factors, it may serve as an alternative method that deserves further empirical evaluation [15].

To determine whether a multiple risk composite should be measured using the reflective or formative indicator approach, Bollen and Diamantopoulos (2017, p. 582) suggest that, “A researcher should imagine a change in the indicator and ask whether this change is likely to change the value of the latent variable. If so, this is theoretical evidence supporting causal or formative indicators.” Applying this standard, we contend that from both a conceptual and methodological perspective, there may be instances in which the formative indicator approach is well aligned with multiple risk assessment. For instance, within the CR perspective, it is conceivable that an increase in maternal education or substance use (i.e., risk indicators) would increase the child’s exposure to CR (i.e., the composite variable). However, CR is not conceived to cause (precede) maternal education or substance use (as is conceptualized with a reflective indicator).

To further clarify the FI approach, we adopt the terminology proposed by Bollen and colleagues [13, 17] to differentiate *causal-formative* and *composite-formative* indicators. We posit that, in many instances, the measurement of a single composite risk variable is theoretically more consistent with the composite-formative indicator method. Unlike the causal FI approach, the composite FI approach does not assume conceptual unity, such that each indicator corresponds with the definition of the concept that the latent variable represents. Furthermore, in contrast to the RI method, the composite FI method does not assume that the individual risk indicators are correlated with one another. Consequently, the composite FI approach is particularly applicable in instances in which investigators seek to derive a single composite risk index to aggregate uncorrelated individual risk indicators.

In contrast to other approaches which make assumptions about the equal weights of the individual risk indicators on the composite measure, one of the potential advantages of the RI and FI methods is that this assumption can be tested empirically comparing two nested measurement models (e.g., via a chi-square difference test). In one model, which assumes unequal weights, an *unconstrained measurement model* can be specified in which factor loadings or coefficients are estimated freely for each indicator. In a second model, which assumes equal weights, a *constrained measurement model* can be specified in which factors loadings (or coefficients) are estimated to be equal to one another. Given that these two models are nested, a chi-square difference test (or likelihood ratio test statistic) can then be used to compare these models and to determine whether imposing equality of weights across indicators results in a reduction in model fit.

### Person-centered methods

CR can also be conceptualized and measured using *person-centered* methods [18]. Person-centered methods such as latent class analysis (LCA) and latent profile analysis (LPA) consist of identifying groups of individuals who exhibit a similar pattern of responses on a pre-specified set of variables. This methodology can be applied to dichotomous risk indicators (using LCA) and to continuous risk indicators (using LPA). That is, the primary distinction between LCA and LPA is the use of categorical or continuous indicators [19]. This methodology can be conceived as a data-driven approach such that the qualitative nature of the identified groups (i.e., latent classes) are not specified a priori, but rather depend on the extent to which the estimated model represents the observed data. Applying this approach to the investigation of multiple risk indicators, it is conceivable that there are subgroups of individuals who exhibit similar risk profiles (e.g., high risk across multiple indicators, low risk across multiple indicators, or a combination of high and low risk across indicators).

There are several considerations in applying person-centered methods for investigating multiple risk. One potential advantage of this approach is that it may provide greater specification about the constellations of risk that are most problematic for adjustment, rather than the total number of risk factors (based on the CR index) or the severity of aggregated risk exposure (based on the PCR or ZCR). In this respect, person-centered methods rely on a markedly different conceptual assumption about how risk exposure impacts adjustment [18]. Stated differently, whereas the CR index assumes that the number of risk factors to which an individual is exposed influences adjustment outcomes, person-centered approaches may provide greater insights—depending on the qualitative distinctions of the latent classes that are identified—into how a specific set of risk factors are associated with adjustment. Because this is a data-driven approach which examines profiles of risk within a given sample, the nature of the latent classes that are identified are sample-specific. Moreover, this approach may require larger sample sizes to distinguish several distinct risk classes (or subgroups).

### Study aims

In order to assess each of the alternative methods described, this study consisted of five primary aims. Aim 1 was to examine the effects of the independent (non-aggregated) risk indicators on children’s externalizing problems. Aim 2 was to examine the simultaneous (additive) effects of the individual risk indicators. Taken together, these two aims were important preliminary steps considering that the primary rationale of forming a composite variable is that it presumably has a stronger association with the outcome variable than any individual risk indicator. Aim 3 was to compute three observed score CR risk indices based on the three sets of individual risk indicators (i.e., dichotomous, proportion- and z-scores). Aim 4 was to incorporate variable-centered analyses as an alternative to the observed score CR indices. Towards this end, the reflective and formative indicator approaches (i.e., RI and FI methods) were examined. Aim 5 was to incorporate person-centered methods, via the use of latent class and latent profile analyses. LCA was used to estimate the models using dichotomous indicators and LPA was used for the proportion- and z-score indicators.

Before addressing these primary aims, numerous studies were reviewed to identify a set of empirically derived risk indicators. Although researchers have not consistently measured the same risk indicators across studies, there are several that have been identified across multiple investigations including: low maternal education, hunger, meal and money unpredictability, maternal psychopathology, maternal substance (e.g., alcohol, tobacco and cocaine use), harsh parenting and discipline, family stress, and family violence [20–29]. Notably, some of these studies have also incorporated measures of family income, race, and family structure; however, because the majority of participants in the current sample were low-income, African American, and single-mothers, these indicators were not included because they exhibited low variability (see Method section for more details on the sample).

For each of the primary aims, effect sizes (*R*^*2*^) were used to compare the relative predictive power of each method. Since one of the primary aims of studies which incorporate a multiple risk variable is to predict a specific outcome of interest, we evaluate the predictive utility of each method on children’s externalizing problems in kindergarten. The rationale for this selected outcome and developmental period was based on several considerations. There is a substantial body of research which has been interested in examining how early childhood risk and adversity impacts children’s psychological adjustment, and in particular the development of externalizing problems (e.g., aggression, disruptive and conduct problems; [3,4,30–34]. In turn, children with higher rates of externalizing problems lack the prerequisite (i.e., school readiness) skills to more effectively adapt to the demands of being in a structured kindergarten classroom environment, increasing their risks for social and academic problems during this important transitional period [31,32]. Consequently, an examination of the role of CR on children’s externalizing problems during this developmental period may serve as an empirical example that is of interest to researchers across multiple disciplines.

## Method

### Participants and procedures

The sample for the current study consisted of 169 parent–child dyads participating in an ongoing longitudinal study. All families were recruited from two urban hospitals serving large numbers of low-income, minority families. The study received approval from the institutional review boards of the hospitals as well as the primary institution at which the study was conducted. Mothers were screened after delivery for initial eligibility in order to identify a sample of participants with high rates of prenatal substance use (for a more detailed explanation of the recruitment and screening procedures, see [35]). Interested and eligible mothers were given detailed information about the study and asked to sign consent forms.

Among recruited mothers who agreed to participate (*N* = 216), 116 had some level of prenatal cocaine use. Participating mothers ranged in age from 18 to 42 years (*M* = 29.78; *SD* = 5.46). The majority of mothers were African-American (74%), were receiving Temporary Assistance for Needy Families (71%) at the time of their first laboratory visit, and were single (60%). Of the 216 children, 106 (49%) were boys. For the aims of this study, the kindergarten wave assessment was used, when children were approximately 5 years old. Measures were derived from a combination of maternal interviews and observations of mother–child interactions. Because of the longitudinal design, participant attrition increased with the passage of time. By the kindergarten wave, 169 participants were actively involved in the project, and 47 participants (21.8%) had dropped out of the study.

### Measures

#### Meal and money unpredictability

Meal and money unpredictability were assessed using the meal and unpredictability subscales from the Family Unpredictability Scale [36]. Meal unpredictability was based on 5 items and money unpredictability was based on 3 items. Respondents rated items on a 5-point scale (*1 = Not at all and 5 = extremely*) and subscales were computed by taking the average score across the indicators (alpha = .60 for meal unpredictability and alpha = .70 for money unpredictability). A proportion score was then created by dividing each respondent’s subscale scores by the maximum score. A standardized score was computed based on the mean scores. A dichotomized score was estimated by identifying the families with some degree of meal and money unpredictability (i.e., average scores greater than 1.5; about 19.5% of families had meal unpredictability and 30.5% had money unpredictability). Descriptive statistics for the individual risk indicators are reported in Table 1.

**Table 1:** Descriptive statistics for individual risk indicators.

		Dichotomized scores				Proportion scores				Z-scores			
	*N*	Min.	Max.	*M*	*SD*	Min.	Max.	*M*	*SD*	Min.	Max.	*M*	*SD*
Money unpredictability	164	0.00	1.00	0.30	0.46	0.00	1.00	0.27	0.25	-1.12	2.95	0.00	1.00
Meal unpredictability	164	0.00	1.00	0.20	0.40	0.00	1.00	0.33	0.23	-1.44	2.88	0.00	1.00
Hunger problems	166	0.00	1.00	0.27	0.45	0.00	1.00	0.14	0.26	-0.52	3.34	0.00	1.00
Family stress	164	0.00	1.00	0.15	0.35	0.00	1.00	0.13	0.17	-0.74	5.17	0.00	1.00
Family violence	167	0.00	1.00	0.15	0.36	0.00	1.00	0.08	0.20	-0.48	5.91	0.00	1.00
Harsh discipline	163	0.00	1.00	0.29	0.45	0.00	1.00	0.13	0.19	-0.66	4.53	0.00	1.00
Low maternal sensitivity	166	0.00	1.00	0.25	0.43	0.01	1.00	0.43	0.24	-1.78	2.38	0.00	1.00
Maternal psychopathology	166	0.00	1.00	0.22	0.41	0.00	1.00	0.15	0.20	-0.73	4.27	0.00	1.00
Maternal cocaine use	167	0.00	1.00	0.08	0.27	0.00	1.00	0.04	0.17	-0.24	5.48	0.00	1.00
Maternal alcohol use	167	0.00	1.00	0.10	0.30	0.00	1.00	0.06	0.18	-0.32	5.26	0.00	1.00
Maternal tobacco use	167	0.00	1.00	0.19	0.39	0.00	1.00	0.10	0.15	-0.67	5.91	0.00	1.00
Low maternal education	169	0.00	1.00	0.27	0.45					-0.61	1.63	0.00	1.00

#### Hunger problems

Hunger problems were assessed by using the Community Childhood Hunger Identification Project (CCHIP) scale [37]. The CCHIP scale consists of 8 items with a dichotomous (yes, no) response choice which reflect hunger problems over the past 12 months. The individual items were summed to create an overall hunger score with total scores ranging from 0–8 (alpha = .90). This measure has been validated in several previous studies on childhood hunger [38,39]. The summed scores were converted into proportion (by dividing by the maximum hunger score), and standardized scores. A dichotomized score was estimated by identifying the families who indicated any degree of hunger problems (27.1%).

#### Maternal psychopathology

The Brief Symptom Inventory (BSI) is a brief form of Symptom Checklist 90-R and is a widely used mental health screening measure in a variety of clinical and research settings [40]. It consists of 53 items rated on a five-point scale which assess a range of psychopathology symptoms (e.g., depression, anxiety, psychoticism, and hostility). A global severity index was computed by taking the average score across all of the 53 items (alpha = .98). Dichotomized scores were based on identifying participants who had a normed T-Score equal to or greater than 60 (21.7%), indicative of participants with scores one SD above the population mean.

#### Maternal substance (alcohol, tobacco and cocaine) use

The Timeline Follow-Back Interview (TLFB) was used to assess self-reported maternal substance use including alcohol, tobacco and cocaine [41]. Participants were provided a calendar and asked to identify events of personal interest (i.e., holidays, birthdays, vacations) as anchor points to aid recall. This method is established as a reliable and valid method of obtaining longitudinal data on substance-use patterns, has good test–retest reliability, and is highly correlated with other intensive self-report measures [42]. The TLFB yielded data about the number of days of binge drinking (defined as having 5 or more standard drinks per day) and cocaine use, and average number of cigarettes smoked per day. For each substance, proportion and standardized scores were computed based on the total number of days used, adjusting for outliers. Dichotomized scores were computed by identifying any degree of cocaine use in the past six months (7.8%), binge drinking at least twice per month (on average) over the past six months (10.2%), and smoking an average of 10 cigarettes or more per day (19.2%).

#### Maternal sensitivity

Maternal sensitivity was measured using behavioral observations during laboratory assessments. Mothers and children were asked to work on a craft project (decorating a picture frame) for 20 minutes [43]. These interactions were coded using the Parent Child Early Relational Assessment (PCERA; [44–46]) by two sets of trained coders blind to any information regarding the families. Coders used a collection of 5-point rating scales to assess the intensity, duration and frequency of specific behaviors (i.e., a score of 1 being equal to a complete lack of, or minimal evidence for, the quality being rated, and a score of 5 being equal to an intense, consistent, or extreme reaction). Inter-rater reliability conducted on a random selection of 14% of the tapes was *r* = .94. Scale scores were derived from taking the average of the coded scores such that higher scores represented lower maternal sensitivity. These scores were transformed into proportion and standardized scores, and to create a dichotomous score, the top quartile (25%) were coded as being high risk.

#### Harsh discipline (corporal punishment)

Harsh discipline was measured by the 5-item physical assault subscale of the Conflict Tactics Scale (CTS; [47]). Mothers reported the frequency (chronicity) with which they engaged in physical discipline (e.g., hitting, slapping or spanking their child) in the past year (alpha = .73). Proportion and standardized scores were computed based on the average scores across the subscale items. A dichotomous score was computed by identifying the participants (28.8%) who had experienced physical discipline in the past year.

#### Family stress

The 31-item Life Experiences Survey was used to assess family stress [48]. Respondents indicated whether they had experienced certain major life events (e.g., death of a family member, serious illness, loss of employment, etc.), and if so, the extent to which the event had negatively impacted their life (*1 = somewhat bad* to *3 = extremely bad*). Item scores were averaged together (alpha = .73). Proportion and standardized scores were computed based on the average scores. A dichotomous measure was computed by identifying the participants (14.6%) who had indicated experiencing a negative impact from these stressful events (i.e., mean scores greater than .25).

#### Family violence

Family violence was assessed by items taken from the TLFB and the CTS [47]. An adapted version of the TLFB was used to measure episodes of family violence. Using a daily calendar over the past six months, mothers were asked about their witnessing, experiencing, or perpetrating violence with their domestic partners. A count variable was computed based on the number of incidents they had indicated. This count variable was used to compute proportion and standardized scores, after adjusting for outliers. A dichotomous score was estimated by identifying the families who indicated any degree of family violence (28.7%). Mothers also completed the Conflict Tactics Scale. The physical assault subscale was used which consisted of 12 items measuring the chronicity that mothers experienced severe and minor forms of physical violence perpetrated by a partner (alpha = .87). This measure was transformed into proportion and standardized scores, and a dichotomous score was estimated by identifying mothers who indicated any degree of physical assault (17.9%). The proportion, standardized, and dichotomous scores for family violence were estimated by aggregating the TLFB and CTS measures. The dichotomous score was computed to identify mothers who had indicated family violence on both measures (15.0%).

#### Maternal education

Mothers were asked to report their highest level of education and a dichotomous variable was created to reflect those who had not completed high school (27.2%). Because this variable aimed to differentiate parents who had not completed high school from those that did, a separate proportion-score for this variable was not estimated (e.g., based on years of schooling). However, in order to maintain a consistent set of indicators across the different methods that were assessed, the dichotomous score was also included in the analyses with proportion-score indicators. For the analyses with standardized scores, a z-score was computed for maternal education based on the dichotomous variable.

#### Externalizing problems

Maternal reports of children’s externalizing problems were obtained using the Child Behavior Checklist (CBCL; [49]). The 19-item aggression subscale was used (alpha = .91), which consists of items reflecting children’s behavioral and emotional problems. Responses options were scored on a 3-point response scale (1 = “not true” to 3 = “very true”).

### Data analysis plan

The analysis plan revolved around the five primary aims. To reiterate, Aims 1 and 2 were to examine the independent and additive effects of individual risk indicators on children’s externalizing problems. Aim 3 was to compute the three observed score CR indices based on the three sets of individual risk indicators (i.e., dichotomized, proportion- and z-scores), and to compare their associations with children’s externalizing problems. These first three aims were addressed using regression analyses. Aim 4 was to incorporate variable-centered latent variable analysis as an alternative to the CR indices. Towards this end, the reflective and formative indicator approaches (i.e., RI and FI methods) were examined using structural equation modeling (SEM) with full information maximum likelihood (FIML) estimation. In these models, multiple fit indices, including the χ^2^, RMSEA, SRMR, and CFI were used to assess model fit. Aim 5 was to incorporate person-centered methods, via the use of latent class and latent profile analyses. LCA was used to estimate the models using dichotomous indicators and LPA was used for the proportion- and z-score indicators. Latent class and latent profile analysis were specified using maximum likelihood with robust standard errors (MLR) estimation. In order to determine the optimal class solution, multiple fit indices were examined as recommended by methodologists (Collins & Lanza, 2010) including the BIC, AIC, sample-size adjusted BIC (SABIC), and Lo-Mendell-Rubin adjusted Likelihood Ratio Test (LMR-aLRT). Models with smaller AIC, BIC and SABIC values indicate better fitting solutions. Significant *p* values on the LMR-aLRT indicate that a model with *k* classes has better fit to the data than a model with *k– 1* classes. Additionally, entropy and class assignment probabilities were assessed which measure classification precision (values ranging from 0 to 1 with values closer to 1 indicative of greater precision). Across all models, the standardized (*b*) coefficients and effect sizes (*R*^*2*^) were used to compare the predictive power of each approach on children’s externalizing problems. Analyses were performed in MPlus version 8 [50].

## Results

Bivariate correlations among the individual risk indicators are reported in Table 2. Correlations above the main diagonal are estimates for the proportion and z-score indicators and below the main diagonal for dichotomous indicators. Taken together, across the three scoring methods, there were several small to moderate correlations among the risk indicators. The size of these correlations provided support for the premise that many of these risk indicators were associated with each other, but also reflected distinct facets of risk.

**Table 2:** Bivariate correlations among the individual risk indicators.

Variables	1	2	3	4	5	6	7	8	9	10	11	12
1. Money unpredictability		0.36**	0.47**	0.21**	0.05	0.09	-0.04	0.34**	0.18*	0.12	0.10	-0.01
2. Meal unpredictability	0.21**		0.23**	0.10	0.05	0.32**	0.17*	0.20*	0.13	0.38**	0.05	-0.04
3. Hunger problems	0.38**	0.15		0.14	0.04	0.23**	0.00	0.36**	0.22**	0.09	0.04	0.02
4. Family stress	0.14	0.01	0.06		0.20**	0.07	0.08	0.27**	0.10	0.09	0.11	0.11
5. Family violence	0.00	0.02	0.08	0.03		0.06	0.05	0.10	0.07	0.18*	0.04	0.01
6. Harsh discipline	0.08	0.30**	0.11	0.04	0.09		0.20**	0.19*	0.24**	0.22**	0.07	0.00
7. Low maternal sensitivity	-0.03	0.01	-0.10	0.09	0.07	0.15		0.02	0.01	0.13	0.00	0.00
8. Maternal psychopathology	0.19*	0.15	0.34**	0.20*	0.21**	0.12	0.06		0.28**	0.17*	0.15	0.10
9. Maternal cocaine use	0.10	0.08	0.18*	0.13	0.07	0.11	-0.07	0.17*		0.28**	0.10	0.04
10. Maternal alcohol use	0.18*	0.31**	0.11	0.04	0.30**	0.15*	0.17*	0.12	0.20*		0.38**	0.10
11. Maternal tobacco use	-0.02	0.12	0.18*	0.06	0.14	0.08	0.00	0.12	0.14	0.14		0.17*
12. Low maternal education	-0.08	0.01	-0.01	0.09	0.00	-0.02	0.00	0.07	0.02	0.06	0.14	

*Correlations above the main diagonal are for the proportion and z-score indicators*, *and below the diagonal for dichotomous indicators*.

*p < .*05;*

**p < .*01;*

### Independent and non-aggregated additive associations of the individual risk indicators on externalizing problems (aims 1 and 2)

To measure the independent effects of each risk indicator on externalizing problems, regression analyses were performed (see Table 3). The standardized coefficients using the proportion score and z-score indicators were identical. To simplify the presentation of results, these estimates are provided only once, and referred to as the continuous variable indicators. Results indicated that for both the dichotomous and continuous variable indicators, money unpredictability, hunger problems, family stress, harsh discipline, maternal psychopathology, and maternal alcohol and tobacco use were significantly associated with externalizing problems. Meal unpredictability was associated with externalizing problems for the continuous, but not dichotomous, indicator. Effect sizes are reported for each indicator, and ranged from .00 to .06 and .00 to .08 for the dichotomous and continuous variables, respectively.

**Table 3:** Independent and additive associations of the individual risk indicators with externalizing problems.

	Independent Effects				Additive Effects			
	Dichotomous Scores		Continuous Scores		Dichotomous Scores		Continuous Scores	
	*B*	*R*^*2*^	*B*	*R*^*2*^	*B*	*R*^*2*^	*B*	*R*^*2*^
Money unpredictability	0.23**	0.05	0.27***	0.07	0.14		0.19*	
Meal unpredictability	0.12	0.02	0.19*	0.04	-0.03		-0.02	
Hunger problems	0.24***	0.06	0.19**	0.04	0.11		0.01	
Family stress	0.17*	0.03	0.25***	0.06	0.11		0.16*	
Family violence	0.12	0.01	0.07	0.01	0.03		-0.02	
Harsh discipline	0.21**	0.04	0.28***	0.08	0.15		0.23**	
Low maternal sensitivity	0.07	0.01	0.08	0.01	0.03		0.02	
Maternal psychopathology	0.23**	0.05	0.24***	0.06	0.11		0.09	
Maternal cocaine use	0.08	0.01	0.06	0.00	-0.04		-0.11	
Maternal alcohol use	0.21**	0.04	0.20**	0.04	0.12		0.10	
Maternal tobacco use	0.15*	0.02	0.17*	0.03	0.09		0.07	
Maternal education	0.06	0.00			0.03		0.01	
Total						0.17		0.21

Note: Results using the proportion score and z-score indicators were identical. To simplify the presentation of results, these estimates are provided only once (referred to above as continuous scores).

*p < .05

**p < .01

***p < .001

Multiple regression was used to estimate the additive effects of each risk indicator controlling for the effects of other indicators (see Table 3). Results using the proportion score and standardized score indicators were identical, and to simplify the presentation of results, these estimates are provided only once (referred to as continuous variable indicators). Among the dichotomous indicators, the results indicated that none of the independent effects were significantly associated with externalizing problems. Among the continuous indicators, money unpredictability, family stress, and harsh discipline were significantly associated with externalizing problems, controlling for the effects of the other indicators. The effect size for the dichotomous indicators was *R*^*2*^ = .17, and for the continuous indicators, *R*^*2*^ = .21, indicating that the additive models yielded effect sizes that were more than twice the magnitude of any of the individual indicators assessed independently (i.e., in the non-aggregated models).

### Observed score methods (aim 3)

#### Cumulative risk (CR) index

**Fig 2:**
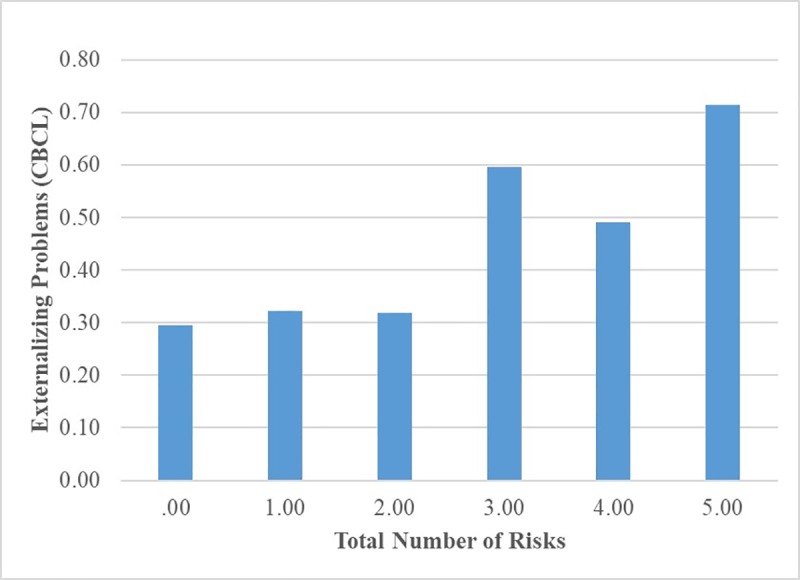
Children’s externalizing problems by total number of risks (based on dichotomous indicators).

To compute the CR index, the dichotomous individual risk indicators were summed together, ranging from 0 to 9 risks (*M* = 2.41, *SD* = 1.97). About 17.2% of children had zero risks, 21.3% had 1 risk, 20.7% had 2 risks, 12.4% had 3 risks, 16.0% had 4 risks and 12.4% had 5 or more risks. The CR index was significantly associated with externalizing problems (*B* = .38, *p* < .001, *R*^*2*^ = .14). To further assess whether there was a threshold effect, mean scores for externalizing problems were compared for children with varying risk. Children with 5 or more risks were combined into one group to account for small frequencies in these groups. The results did not consistently indicate the presence of a threshold effect (see Fig 2). Post-hoc analyses revealed that the children with 3 or 5 or more risks had higher levels of externalizing problems than children with 0, 1 or 2 risks, but these differences were not statistically significant for children with 4 risks.

#### Proportion-score cumulative risk (PCR) index

To compute the PCR index, the proportion score individual risk indicators were averaged (*M* = .18, *SD* = .10, range = .00 to .57). The PCR index was significantly associated with externalizing problems (*B* = .36, *p* < .001, *R*^*2*^ = .13).

#### Standardized (z-score) cumulative risk (ZCR) index

To compute the ZCR index, the standardized individual risk indicators were averaged (*M* = .00, *SD* = .46, range = -.75 to 2.13). The ZCR index was significantly associated with externalizing problems (*B* = .38, *p* < .001, *R*^*2*^ = .14).

### Variable-centered methods (aim 4)

#### Reflective indicator (RI) method

**Fig 3:**
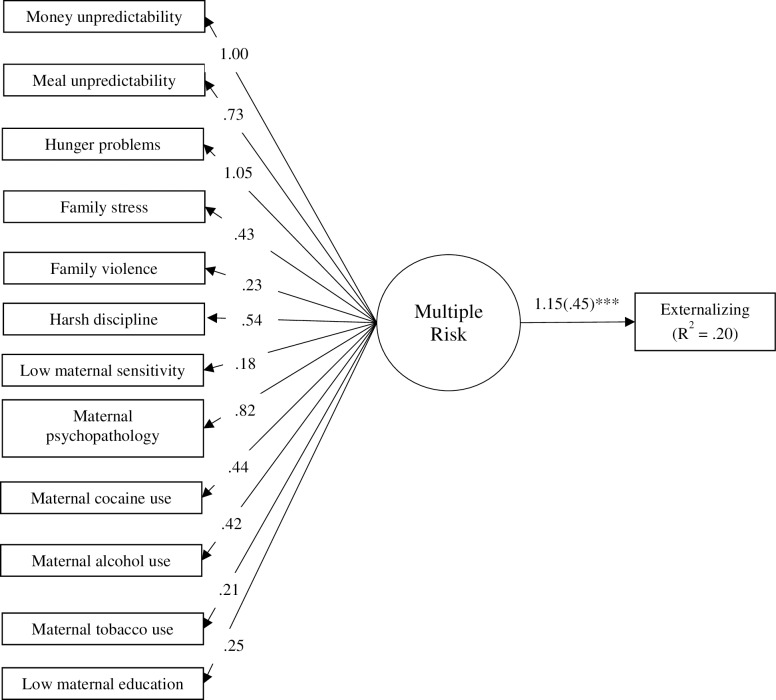
Path diagram for reflective indicator (RI) method examining the associations of multiple risk indicators (proportion scores) on children’s externalizing problems. Unstandardized estimates are provided, and for the effect on externalizing problems, the standardized estimate is shown in parentheses. ***p < .001.

Structural equation modeling (SEM) with maximum likelihood estimation was used to specify latent variable models to assess the RI method. Separate models were specified using the varying individual risk indicators (i.e., dichotomous, proportion and standardized scores). Using the proportion score indicators, a latent variable model was specified in which the 12 individual risk indicators were used to specify a multiple risk latent construct (see Fig 3). Residual covariances were estimated for indicators that were derived from the same measure (e.g., between meal and money unpredictability, and among maternal alcohol, tobacco and cocaine use), but are not shown in Fig 3 to simplify the presentation of results. The factor loadings for this model were unconstrained, indicating that the individual risk indicators had unequal weights on the latent construct. This model had inadequate model fit (*χ*^*2*^ = 93.36, *df* = 58, *p* < .01; RMSEA = .06, SRMR = .06, CFI = .84) and there were considerable variations in the size of the factor loadings across the indicators. It appeared that the loadings for family violence, maternal sensitivity, maternal tobacco use and maternal education were particularly low (i.e., less than .40). The latent construct reflecting multiple risk was a significant predictor of externalizing problems (*B* = .45, *p* < .001, *R*^*2*^ = .20). Using the same parameters, this model was specified again using the standardized scores. Notably, results across these models were identical, with the exception of slight variations in the unstandardized factor loadings.

By permitting the factor loadings to vary across indicators this model did not assume that each risk indicator would have an equal weight on the latent factor. However, because the overall model fit was low, it was not feasible to impose equal weights (i.e., constrain factor loadings) as this approach would further reduce fit. Thus, a constrained model was not specified.

To examine whether the use of the RI method varied by using dichotomous scores, the latent variable model was re-specified using the WLSMV estimator to accommodate for categorical data. Similar to the models with proportion/standardized scores, there was considerable variation in the size of the factor loadings across the indicators (i.e., the unstandardized factor loadings ranged from .15 to 1.19). The latent construct reflecting multiple risk was a significant predictor of externalizing problems (*B* = .46, *p* < .001, *R*^*2*^ = .21).

#### Formative indicator (FI) method

Path analysis was used to specify the FI method. In contrast to the RI method in which the individual risk indicators are reflective indicators of the latent construct, in the FI method, the indicators are conceived as composite formative indicators. Stated differently, the arrows in the path model are from the indicators to the composite variable (as opposed to the RI method in which the paths are from the latent construct to the indicators).

**Fig 4:**
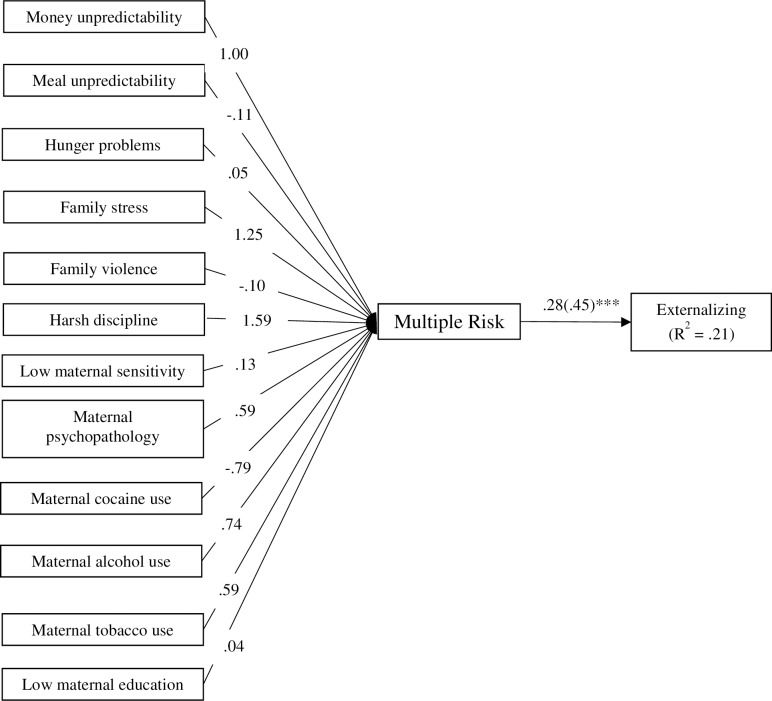
Path diagram for formative indicator (FI) method examining the associations of multiple risk indicators (proportion scores) on children’s externalizing problems. Unstandardized estimates are provided, and for the effect on externalizing problems, the standardized estimate is shown in parentheses. ***p < .001.

Using the proportion score indicators, a model was specified in which the 12 individual risk indicators were used to predict a multiple risk composite variable (see Fig 4). To specify the composite variable, its variance was constrained to zero [12]. The composite multiple risk variable was a significant predictor of externalizing problems (*B* = .45, *p* < .001, *R*^*2*^ = .21). Using identical parameters, this model was specified again using the standardized scores. Notably, results across these models were identical, with the exception of slight variations in the unstandardized factor loadings. To examine whether the use of the FI method varied by using the dichotomous individual risk indicators, the model was specified again using the dichotomous scores. The multiple risk composite variable was a significant predictor of externalizing problems (*B* = .41, *p* < .001, *R*^*2*^ = .17).

### Person-centered methods (aim 5)

#### Latent class analysis (LCA)

**Fig 5:**
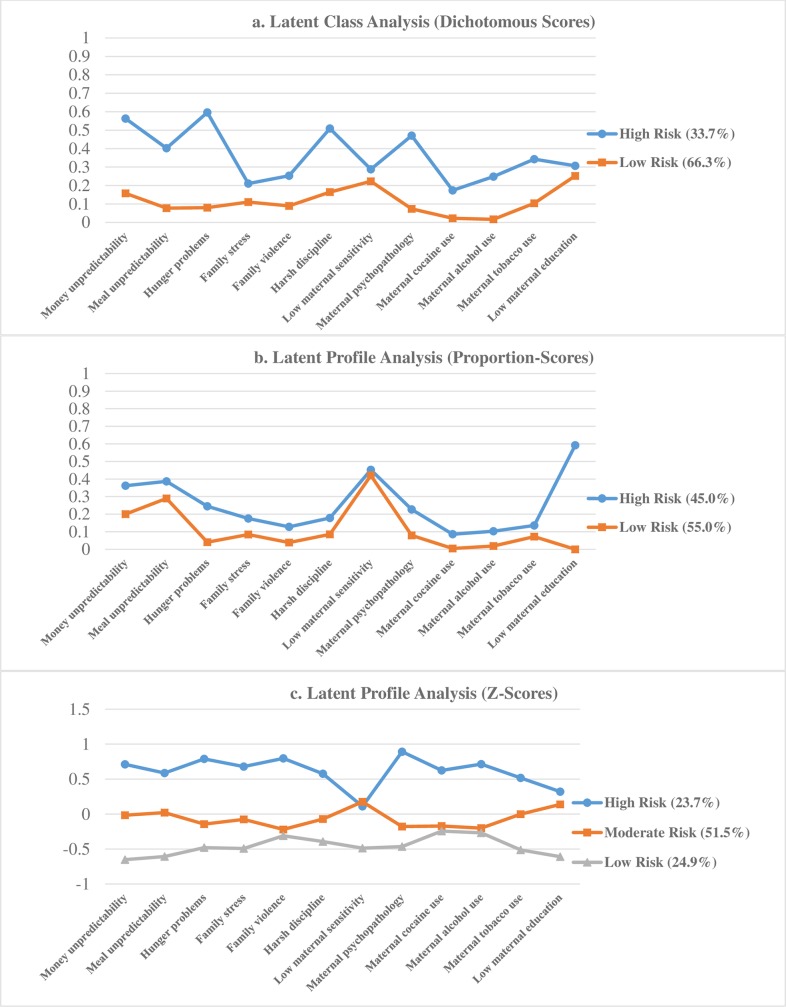
Results for person-centered methods (latent class and profile analyses) which identified multiple risk classes based on 12 individual risk indicators (derived from dichotomous, proportion- and z-scores).

Using the dichotomous scores, a series of LCA models were specified starting with a 2-class solution and introducing additional classes (i.e., 3- thru 5- classes). Class-specific probabilities were plotted to assess the qualitative nature of the classes and to determine whether each class was conceptually meaningful and interpretable. Based on evaluating the fit indices and the qualitative nature of the classes, the 2-class solution was determined to be the optimal model solution. Compared to models with varying numbers of classes, this model had the lowest BIC, and a statistically significant LMR-aLRT (see Table 4). This model consisted of two distinct classes, one characterized as being a *low-risk* class (66.3%), and the second being a *high-risk* class (33.7%). Class-specific probabilities are shown in Fig 5A. Notably, estimating a 5-class model resulted in convergence problems and the model not replicate consistently, indicating a possible local maxima [19]. Therefore, fit indices for this model are not reported in Table 4.

**Table 4:** Model fit indices for latent class and profile analyses.

Model	LogL	AIC	BIC	SABIC	Entropy	LMR-aLRT
LCA (Dichotomous scores)						
**Two-class**	**-933.47**	**1916.95**	**1995.19**	**1916.04**	**0.65**	84.24**
Three-class	-917.71	1911.43	2030.36	1910.04	0.80	31.05
Four-class	-905.77	1913.55	2073.17	1911.69	0.79	23.53
LPA (Proportion scores)						
**Two-class**	**421.48**	**-788.96**	**-704.45**	**-789.94**	**0.92**	723.31**
Three-class	599.65	-1117.29	-988.97	-1118.79	0.94	351.77
Four-class	715.38	-1320.75	-1148.61	-1322.76	0.95	228.67
Five-class	785.41	-1432.82	-1216.86	-1435.33	0.94	138.56
LPA (Z-scores)						
Two-class	-2526.43	5106.85	5191.36	5105.87	0.86	574.49***
**Three-class**	**-2407.70**	**4897.39**	**5025.72**	**4895.90**	**0.89**	234.20*
Four-class	-2350.96	4811.92	4984.06	4809.92	0.91	112.02

Note. LogL = Loglikelihood, AIC = Akaike information criteria, BIC = Bayesian information criteria, SABIC = Sample-size adjusted Bayesian information criteria; LMR-aLRT = Lo Mendell Rubin adjusted likelihood ratio test, LCA = Latent class analysis, LPA = latent profile analysis. Rows in bold were selected as the optimal model solution. In the LCA and LPA (z-score) models, estimating a 5-class model resulted in convergence problems and the model not replicate consistently. Therefore, fit indices for these models are not reported above.

*p < .05

**p < .01

***p < .001

#### Latent profile analysis (LPA)

Similar to the LCA, a series of LPA models were specified using the proportion and z-score indicators. With respect to the proportion score indicators, although the information criteria (AIC, BIC, and SABIC) consistently favored models with increasing numbers of classes, the LMR-aLRT indicated that the 2-class solution had a better fit than the 1-class solution, but additional classes did not significantly improve model fit (see Table 4). Based on these considerations, the 2-class model was selected as the optimal solution. This model (see Fig 5B) consisted of a *high-risk* class (45.0%), and a *low-risk* class (55.0%).

Using the z-score indicators, results for the LPA models indicated that the information criteria (AIC, BIC, and SABIC) consistently favored models with increasing numbers of classes. However, the LMR-aLRT indicated that the 3-class solution had better fit than the 2-class solution, but the 4-class solution did not have significantly better fit than the 3-class solution (see Table 4). Based on these considerations, the 3-class model was selected as the optimal solution. This model consisted of a *high-risk* class (23.7%), a *moderate-risk* class (51.5%) and a *low-risk* class (24.9%). Class-specific means are shown in Fig 5C. Notably, estimating a 5-class model resulted in convergence problems and the model not replicate consistently. Therefore, fit indices for this model are not reported in Table 4.

#### Comparing LCA and LPA models

Comparing the solutions across the LCA and LPA models demonstrated that the use of dichotomous, z-score and proportion-score indicators appeared to influence class identification. To further assess how consistently these models differentiated children who could be characterized as high-risk, the individual class assignment probabilities derived from these models were extracted. Bivariate correlations revealed that across the models the probability of being high-risk ranged from *r* = .47 to .63. Thus, although the results indicated moderate to high consistency across the three models with respect to children’s classifications, some inconsistencies also emerged across these methods.

To assess the associations of being in the high-risk class and externalizing problems, a series of regression analyses were performed. More specifically, for each model that was selected based on the dichotomous, z-score, and proportion-score indicators, children’s class assignment probabilities of being in the high-risk class were used to predict externalizing problems. Notably, in the LCA/LPA framework, there are different approaches to examine the associations between class membership and outcome variables, however, this particular approach was selected because it provided standardized regression coefficients (*B*) and effect size measures (*R*^*2*^) that were directly comparable to the other multiple risk methods assessed.

Using the class assignment probabilities from the LCA (i.e., using dichotomous indicators), the results indicated that children who were more likely to be classified in the high-risk class had significantly higher rates of externalizing problems (*B* = .37, *p* < .001; *R*^*2*^ = .14). Results from the LPA model using the z-score indicators (*B* = .25, *p* < .001; *R*^*2*^ = .06) and proportion score indicators (*B* = .24, *p* < .001; *R*^*2*^ = .06) also indicated that children who were classified in the high-risk classes had significantly higher rates of externalizing problems. However, the effect sizes for these models were lower compared to the LCA model.

## Discussion

This study contributes to extant research in several ways. Perhaps most importantly, it provides a comprehensive assessment of alternative methods to measure cumulative risk (CR) based on multiple risk indicators, elaborating on their conceptual assumptions and operationalization. Although many studies have incorporated CR variables, it has been rare for investigators to compare multiple methods systematically [3]. To our knowledge, this is the first study to attempt an empirical comparison across the seven proposed methods. A decision tree (Fig 1) was proposed to aid researchers in determining which methods may be more appropriate in consideration of, 1) the measurement and operationalization of the individual risk indicators, 2) the overall analytic design and feasibility of using observed or latent scores, and 3) how the primary research objectives align with variable- or person-centered methods. In scenarios in which investigators opt to use variable-centered analyses, we clarified the rationale for using the reflective or (composite) formative indicator methods.

### Independent and additive (non-aggregated) associations of the individual risk indicators on externalizing problems

The first aim of this study was to examine the effects of the independent (non-aggregated) risk indicators on children’s externalizing problems. These analyses provided a baseline by which to assess whether the aggregation of multiple risk indicators had a stronger association with externalizing problems than any single indicator. Although this is a longstanding assumption of the CR approach [1, 3, 51], we contend that testing this assumption empirically should serve as an integral preliminary step when investigators are determining to utilize a CR measure. The results indicated that several of the individual risk indicators including money unpredictability, hunger problems, family stress, harsh discipline, and maternal psychopathology, alcohol and tobacco use were significantly associated with children’s externalizing problems. Moreover, consistent with CR perspectives [1, 3, 51], compared to the individual risk indicators, the effect sizes for the CR indices clearly indicated that they provided a more robust measure which was more strongly associated with externalizing problems.

Although an examination of the individual risk indicators provided some insights into whether the different approaches to operationalizing these indicators—that is dichotomous, proportion-, or z-scores—impacted their strength of association with externalizing problems, decisions pertaining to operationalization must also take into account practical and conceptual considerations. On the one hand, methodologists have raised concerns about the potential impact of dichotomizing continuous variables [8,9]. On the other hand, it is common practice in many areas of psychological research to dichotomize variables to select out more severe cases of risk [3].

The second aim of this study was to perform multiple regression analyses to assess the simultaneous (additive) effects of the individual risk indicators. Findings validated the premise that the additive effects of multiple risks outweighed the independent effects. More specifically, across the three approaches of operationalizing the individual risk indicators, the additive effect sizes were more than twice as large as any of the independent effects. Notably, the additive effects of the continuous scores (i.e., proportion-scores and z-scores) had a slightly larger association with externalizing problems than the dichotomous scores. A comparison of the independent and additive models also raises some questions about potential suppression effects. That is, in contrast to the independent effects which indicated that more than half of the risk indicators were significantly associated with externalizing problems, none of the dichotomous indicators, and three of the continuous indicators had a statistically significant association with externalizing problems, even though the overall effect sizes in the additive models were larger.

### Observed score methods

The third aim of this study was to compute three observed score cumulative risk indices (i.e., CR, PCR and ZCR indices) based on the three sets of individual risk indicators, and to compare their associations with externalizing problems. Results indicated that the differences in effect sizes across these three approaches were fairly negligible. In comparing the PCR and ZCR approaches, one of their fundamental differences was the extent to which any of the individual risk indicators may have a differential impact on the composite index (i.e., the relative weight of each indicator). Along these lines, the results indicated trivial differences between these two approaches, suggesting that they are fairly interchangeable and yield similar effects.

In summary, although decisions about how to operationalize the risk indicators are grounded in different conceptualizations of risk (e.g., measuring risk indicators along a continuum, and differential weight of the risk indicators), it appeared that these distinctions did not have much bearing on the predictive power of the observed score indices. Because relatively few studies have used the PCR or ZCR indices (as opposed to the CR index), comparisons across these approaches have been rare. Thus, additional research evaluating these alternative approaches may yield greater confidence in whether these findings are more broadly generalizable, or specific to the sample, risk indicators, and outcome evaluated in the current study.

It is also worth noting that across these three CR indices, the overall effect sizes were smaller than what was observed with the additive regression analyses, but larger than the independent effects. These results are fairly consistent with those reported by Evans et al. (2013). In their comprehensive review of studies, they found that across 95 comparisons which explicitly compared the CR index to the additive approach (assessing a variety of children’s outcomes including externalizing problems), 58 of these comparisons indicated that the additive approach resulted in a larger effect size, 7 comparisons indicated the CR index resulted in a larger effect size, and 30 comparisons yielded comparable effect sizes.

Beyond examining effect sizes, there may be other considerations for researchers when deciding to use a CR index compared to examining the additive effects of the individual risk indicators. The use of many (in our case 12) individual risk indicators is likely to add considerable complexity to model specification (e.g., the inclusion of many additional parameters within a SEM framework). Furthermore, researchers are likely to face challenges accurately interpreting the results if dealing with potential suppression effects across the individual indicators.

### Variable-centered methods

The fourth aim of this study was to incorporate variable-centered analyses as an alternative to the observed score CR indices. Towards this end, the reflective and formative indicator approaches were examined (i.e., the RI and FI methods). The results indicated that regardless of how the individual risk indicators were operationalized, both of these methods yielded effect sizes that essentially matched, and slightly outperformed the effects observed in the additive models. Consequently, these methods also outperformed the three observed score CR indices as well. Although these methods yielded relatively larger effect sizes, they also raised several caveats that deserve further consideration. Both methods yielded models in which the factors loadings (for the RI models) and the coefficients (in the FI models) resulted in the individual risk indicators having unequal weight on the multiple risk composite. Furthermore, given the wide range in factor loadings in the RI models it appeared that some risk indicators (e.g., family violence, maternal sensitivity, maternal tobacco use and maternal education) had a very small contribution towards the latent construct, even though these were not necessarily the variables that had non-significant associations with externalizing problems. Finally, the overall model fit for the RI model was inadequate. Taken together, these caveats raise conceptual and methodological concerns about the applicability and appropriateness of this approach for assessing CR.

Underlying the decision to use the RI or FI method are assumptions about the nature of associations among the individual risk indicators and the composite variable. The RI method assumes that there is conceptual unity and internal consistency among the indicators. Although this is often the case when specifying latent constructs, this method may not be appropriate to use when this assumption is not reasonably met. Consequently, when the risk indicators lack conceptual unity and internal consistency (reliability), the composite FI method is more suitable [12, 13, 15]. In the current study, it is presumable that the risk indicators were intended to assess risk across multiple domains and did not maintain conceptual unity or internal consistency. Although some were correlated, this was not the case across the collective set of indicators. Thus, it appeared that the composite FI method was better aligned with assessing multiple risk than the RI method. It is important to recognize that this conclusion was based on the operationalization of a *single* composite measure of CR, measuring multiple domains of risk. Indeed, the primary aims of this study were to examine alternative methods for aggregating the individual risk indicators into *one* composite risk variable. However, there may be instances in which investigators are interested in examining the effects of domain-specific risk factors and seek to specify more than one composite risk variable in order to partition the individual risk indicators into *domain-specific* factors.

### Person-centered methods

The fifth aim of this study was to incorporate and assess person-centered methods. LCA was used to estimate the models using dichotomous indicators and LPA was used for the proportion- and z-score indicators. Across these models, it appeared that the different indicators had an impact on class identification and the corresponding fit indices. Notwithstanding these differences, all three models consistently identified a class of children, labelled as *high-risk*, who exhibited relatively higher levels of risk across the collective set of indicators. Results indicated that children in the high-risk classes were at greater risk for having externalizing problems, however there were some variations in the effect sizes corresponding with these models.

From a multiple risk perspective, person-centered methods are typically advantageous when researchers are interested in identifying discrete subgroups of individuals with distinct constellations of risk [18]. Notably, in the current study, the identified classes were characterized by quantitative (i.e., mean-level) differences, and were not qualitatively distinct such that they were higher on some risk indicators, but lower on others. Methodologists have noted that this pattern of findings may be reflective of an underlying multivariate normal distribution, indicating that there are not qualitatively distinct subgroups of individuals in the population [52]. Alternatively, perhaps identifying additional classes of children who are more qualitatively distinct in their risk profiles requires larger sample sizes than was available in the current analyses. If this is in fact the case, this methodology may require the use of larger sample sizes, compared to the other methods that were examined.

### Study limitations and future directions

#### Sample specificity and generalizability of findings

Taken together, the findings provided novel insights into the relative strength of associations among the various CR approaches and children’s externalizing problems. However, there are several limitations to these findings, which have important implications for future research. Perhaps, most importantly, the findings reported in this study are specific to: 1) the collective set of multiple risk indicators, 2) the sample characteristics, and 3) the specific outcome under investigation. Thus, the extent to which the pattern of associations reported in this study would replicate in other samples remains an important empirical question and direction for future research. Additional studies that utilize comparable methods with different populations, and similar (or varying) sets of multiple risk indicators and outcomes, would collectively increase generalizability, and provide insights pertaining to the potential sample-specific variations of the different approaches examined here.

With respect to the first and second points, it is unclear whether the overall pattern of findings was impacted by the specific risk indicators which were included in these models. Although prior research and theory are likely to inform decisions pertaining to which risk indicators to assess, investigators must also consider the demographics of their sample participants. For instance, in the current study, including risk indicators to assess low family income or single parent households would have identified the majority of participants. Thus, including these variables was not likely to yield much advantage in terms of differentiating risk exposure among participants. In addition to the selection of risk indicators, a related issue pertains to the measurement quality of each indicator. Presumably, some indicators are assessed with more valid and reliable measures than others, introducing potential concerns with measurement error. Although it was beyond the scope of this study to explicitly consider the potential implications of measurement quality, this may serve as an interesting direction for future research (e.g., via the use of simulation studies).

With respect to the third point, it is unclear whether the pattern of findings was specific to the outcome investigated, children’s externalizing problems. Although multiple risk measures are robust predictors of children’s adjustment across multiple domains [3], we cannot rule out the possibility that the alternative methods assessed may have differential effects on other outcomes (e.g., internalizing problems or cognitive development), or had externalizing behaviors been measured using a different informant (e.g., teacher reports). Notwithstanding these potential differences, the methods used in this study could be readily applied to simultaneously examine multiple developmental outcomes. Thus, additional research comparing these alternative methods across multiple outcomes (and informants) would provide further insights pertaining to their robustness.

It is also important to note that there are other methods, not examined in the current study, which can be applied to aggregate multiple risk indicators. For instance, other factor analytic methods, principal components analysis, and cluster analysis may have derived complementary findings. Although the methods included in this study were not exhaustive, they collectively reflect a combination of some of the most commonly used methods in prior research [3], as well as some promising alternatives that have been used less frequently.

#### Assessing multiple (domain-specific) risk domains

As previously noted, the approaches used in this study revolved around an examination of *single* composite measures of CR. An interesting direction for future research would be to extend these methods to the examination of multiple, domain-specific risk composite measures. Such an examination would provide complementary insights, and expand on the various conceptualizations of risk exposure that have been investigated in developmental and clinical research on children’s mental health and psychological adjustment. Importantly, one of the strengths of the various approaches reviewed in this study is that they represent flexible methodologies that can be readily extended to the examination of multiple risk domains. By examining additional latent (or composite) factors in the RI and FI methods, and additional latent classes in the LCA and LPA models, each of these approaches can be used to incorporate multiple risk domains. Whereas LCA and LPA reflect a data-driven approach for examining multiple risk domains, the RI and FI methods may require a priori considerations about the specification of multiple risk domains.

Regardless of the approach used, the examination of multiple risk domains is an important step to further elucidate which risk profiles, or constellations of risk, are particularly detrimental for children’s adjustment. Furthermore, such an examination may have implications for better understanding the mechanisms by which risk exposure impacts child development, and intervention efforts that aim to improve children’s developmental trajectories by reducing their exposure to specific risk factors. For instance, some investigators have suggested differentiating risk exposure along two dimensions representing deprivation and threat [53,54]. Although such a reconceptualization would require a shift from examining a single CR composite to examining multiple domains of risk, we contend that the approaches reviewed in this study provide a methodological framework to pursue this interesting line of research.

## References

[1] Rutter M. Protective factors in children's responses to stress and disadvantage. Annals of the Academy of Medicine. 1979;8: 324–338. 547874

[2] Rutter M., Stress coping and development: Some issues and some questions. Journal of Child Psychology and Psychiatry. 1981;22: 323–356. 10.1111/j.1469-7610.1981.tb00560.x 7287844

[3] Evans GW, Li D, Whipple SS. Cumulative risk and child development. Psychological Bulletin. 2013;139: 1342–1396. 10.1037/a0031808 23566018

[4] Evans GW. The environment of childhood poverty. American Psychologist. 2004;59: 77–92. 10.1037/0003-066X.59.2.77 14992634

[5] Wilson MN, Hurtt CL, Shaw DS, Dishion TJ, Gardner F. Analysis and influence of demographic and risk factors on difficult child behaviors. Prevention Science. 2009;10: 353–365. 10.1007/s11121-009-0137-x 19475510 PMC2793541

[6] Cohen J, Cohen P, West SG, Aiken LS. Applied multiple regression/correlation analysis for the behavioral sciences 3rd ed. Mahwah, NJ: Erlbaum; 2003.

[7] Lucio R, Hunt E, Bornovalova M. Identifying the necessary and sufficient number of risk factors for predicting academic failure. Developmental Psychology. 2012;48: 422–428. 10.1037/a0025939 22182300

[8] Cohen J. The cost of dichotomization. Applied Psychological Measurement. 1983;7: 249–253.

[9] MacCallum RC, Zhang S, Preacher KJ, Rucker DD. On the practice of dichotomization of quantitative variables. Psychological Methods. 2002;7: 19–40. 11928888 10.1037/1082-989x.7.1.19

[10] Moran L, Lengua LJ, Zalewski M, Ruberry E, Klein M, Thompson S, et al Variable-and person-centered approaches to examining temperament vulnerability and resilience to the effects of contextual risk. Journal of Research in Personality. 2017;67: 61–74. 10.1016/j.jrp.2016.03.003 28408769 PMC5386509

[11] Burchinal M, Vernon-Feagans L, Cox M, Key Family Life Project Investigators. Cumulative social risk, parenting, and infant development in rural low-income communities. Parenting: Science and Practice. 2008; 8: 41–69. 10.1080/15295190701830672 19920877 PMC2777722

[12] Bollen KA, Bauldry S. Three Cs in measurement models: Causal indicators, composite indicators, and covariates. Psychological Methods. 2011;16: 265–284. 10.1037/a0024448 21767021 PMC3889475

[13] Bollen KA, Diamantopoulos A. In defense of causal-formative indicators: A minority report. Psychological Methods. 2017;22: 581–596. 10.1037/met0000056 26390170 PMC6670294

[14] Brumley LD, Brumley BP, Jaffee SR. Comparing cumulative index and factor analytic approaches to measuring maltreatment in the National Longitudinal Study of Adolescent to Adult Health. Child Abuse & Neglect. 2018; 10.1016/j.chiabu.2018.08.014 30146090

[15] Hall JE, Sammons P, Sylva K, Melhuish E, Taggart B, Siraj‐Blatchford I, et al Measuring the combined risk to young children's cognitive development: An alternative to cumulative indices. British Journal of Developmental Psychology. 2010;28: 219–238. 10.1348/026151008X399925 20481385

[16] Howell RD, Breivik E, Wilcox JB. Reconsidering formative measurement. Psychological Methods. 2007;12: 205–218. 10.1037/1082-989X.12.2.205 17563173

[17] Bollen KA. Evaluating effect, composite, and causal indicators in structural equation models. *Management Information Systems Quarterly*. 2011;35: 359–372.

[18] Lanza ST, Rhoades BL, Nix RL, Greenberg MT, Conduct Problems Prevention Research Group. Modeling the interplay of multilevel risk factors for future academic and behavior problems: A person-centered approach. Development and Psychopathology. 2010;22: 313–335. 10.1017/S0954579410000088 20423544 PMC3005302

[19] Collins LM, Lanza ST. Latent class and latent transition analysis: With applications in the social, behavioral, and health sciences. New York, NY: John Wiley & Sons; 2010.

[20] Copeland W, Shanahan L, Costello EJ, Angold A. Configurations of common childhood psychosocial risk factors. Journal of Child Psychology and Psychiatry. 2009;50: 451–459. 10.1111/j.1469-7610.2008.02005.x 19220623 PMC2685166

[21] Fanti KA, Henrich CC. Trajectories of pure and co-occurring internalizing and externalizing problems from age 2 to age 12: findings from the National Institute of Child Health and Human Development Study of Early Child Care. Developmental Psychology. 2010;46: 1159–1175. 10.1037/a0020659 20822230

[22] Fergusson DM, Horwood LJ, Lynskey M. The childhoods of multiple problem adolescents: A 15‐year longitudinal study. Journal of Child Psychology and Psychiatry. 1994;35: 1123–1140. 10.1111/j.1469-7610.1994.tb01813.x 7995847

[23] Gach EJ, Ip KI, Sameroff AJ, Olson SL. Early cumulative risk predicts externalizing behavior at age 10: The mediating role of adverse parenting. Journal of Family Psychology. 2018;32:92–102. 10.1037/fam0000360 29543487

[24] Jones DJ, Forehand R, Brody G, Armistead L. Psychosocial adjustment of African American children in single‐mother families: A test of three risk models. Journal of Marriage and Family. 2002;64: 105–115. 10.1111/j.1741-3737.2002.00105.x

[25] Kim S, Brody GH. Longitudinal pathways to psychological adjustment among Black youth living in single-parent households. Journal of Family Psychology. 2005;19: 305–313. 10.1037/0893-3200.19.2.305 15982108

[26] Krishnakumar A, Black MM. Longitudinal predictors of competence among African American children: The role of distal and proximal risk factors. Journal of Applied Developmental Psychology. 2002;23: 237–266. 10.1016/S0193-3973(02)00106-5.

[27] Loeber R, Farrington DP, Stouthamer-Loeber M, Moffitt TE, Caspi A, Lynam D. Male mental health problems, psychopathy, and personality traits: Key findings from the first 14 years of the Pittsburgh Youth Study. Clinical Child and Family Psychology Review. 2001;4: 273–297. 10.1023/A:1013574903810 11837460

[28] Rouse HL, Fantuzzo JW. Multiple risks and educational well being: A population-based investigation of threats to early school success. Early Childhood Research Quarterly. 2009;24: 1–14. 10.1016/j.ecresq.2008.12.001

[29] Shaw DS, Winslow EB, Owens EB, Hood N. Young children's adjustment to chronic family adversity: A longitudinal study of low-income families. Journal of the American Academy of Child & Adolescent Psychiatry. 1998;37: 545–553. 10.1097/00004583-199805000-00017 9585657

[30] Moffitt TE. Adolescence-limited and life-course-persistent antisocial behavior: A developmental taxonomy. Psychological Review. 1993;100: 674–701. 8255953

[31] Patterson GR. Coercive family process Vol. 3 Eugene, OR: Castalia; 1982.

[32] Patterson GR, Reid JB, Dishion TJ. Antisocial boys: A social interactional approach Eugene, OR: Castalia; 1992.

[33] Shaw DS, Hyde LW, Brennan LM. Early predictors of boys' antisocial trajectories. Development and Psychopathology. 2012;24: 871–888. 10.1017/S0954579412000429 22781860 PMC3409584

[34] Shaw DS, Shelleby EC. Early-starting conduct problems: Intersection of conduct problems and poverty. Annual Review of Clinical Psychology. 2014;10: 503–528. 10.1146/annurev-clinpsy-032813-153650 24471370 PMC4194898

[35] Eiden RD, Coles CD, Schuetze P, Colder CR. Externalizing behavior problems among polydrug cocaine-exposed children: Indirect pathways via maternal harshness and self-regulation in early childhood. Psychology of Addictive Behaviors. 2014;28: 139–153. 10.1037/a0032632 23647157 PMC4174429

[36] Ross LT, Hill EM. The family unpredictability scale: Reliability and validity. Journal of Marriage and Family. 2000;62: 549–562.

[37] Wehler CA, Scott RJ, Anderson JJ. Community Childhood Hunger Identification Project: A Survey of Childhood Hunger in the United States. Washington DC: Food Research and Action Center; 1991.

[38] Wehler C, Weinreb LF, Huntington N, Scott R, Hosmer D, Fletcher K, Goldberg R, Gundersen C. Risk and protective factors for adult and child hunger among low-income housed and homeless female-headed families. American Journal of Public Health. 2004;94: 109–115. 10.2105/ajph.94.1.109 14713707 PMC1449835

[39] Murphy JM, Wehler CA, Pagano ME, Little M, Kleinman RE, Jellinek MS. Relationship between hunger and psychosocial functioning in low-income American children. Journal of the American Academy of Child & Adolescent Psychiatry. 1998;37: 163–170. 10.1097/00004583-199802000-00008 9473912

[40] Derogatis LR. Brief Symptom Inventory: Administration, scoring, and procedures manual. Minneapolis, MN: National Computer Systems (NCS); 1993.

[41] Sobell MB, Sobell LC, Klajner F, Pavan D, Basian E. The reliability of a timeline method for assessing normal drinker college students' recent drinking history: Utility for alcohol research. Addictive behaviors. 1986;11: 149–161. 3739800 10.1016/0306-4603(86)90040-7

[42] Brown RA, Burgess ES, Sales SD, Whiteley JA, Evans DM, Miller IW. Reliability and validity of a smoking timeline follow-back interview. Psychology of Addictive Behaviors. 1998;12: 101–112. 10.1037/0893-164X.12.2.101

[43] Kochanska G, Murray KT. Mother–child mutually responsive orientation and conscience development: From toddler to early school age. Child Development. 2000;71: 417–431. 10834474 10.1111/1467-8624.00154

[44] Clark R. The parent-child early relational assessment: A factorial validity study. Educational and Psychological Measurement. 1999;59: 821–846. 10.1177/00131649921970161

[45] Clark R, Hyde JS, Essex MJ, Klein MH. Length of Maternity Leave and Quality of Mother‐Infant Interactions. Child Development. 1997;68: 364–383. 9180007 10.1111/j.1467-8624.1997.tb01945.x

[46] Clark R, Paulson A, Conlin S. Assessment of developmental status and parent-infant relationships: The therapeutic process of evaluation In: Zeanah C, editor. Handbook of infant mental health. New York: Guilford; 1993 pp. 191–209.

[47] Straus MA, Hamby SL, Boney-McCoy S, Sugarman DB. The revised conflict tactics scales (CTS2) development and preliminary psychometric data. Journal of Family Issues. 1996;17: 283–316.

[48] Sarason IG, Johnson JH, Siegel JM. Assessing the impact of life changes: development of the Life Experiences Survey. Journal of consulting and clinical psychology. 1978;46: 932–946. 701572 10.1037//0022-006x.46.5.932

[49] Achenbach TM. Manual for the Child Behavior Checklist/2-3 and 1992 profile. Burlington, VT: Department of Psychiatry, University of Vermont: 1992.

[50] Muthen LK, Muthen BO. Mplus User’s Guide. 8th ed. Los Angeles, CA: Muthén & Muthén; 2017.

[51] Sameroff AJ, Seifer R, Zax M, Barocas R. Early indicators of developmental risk: Rochester Longitudinal Study. Schizophrenia Bulletin. 1987;13: 383–394. 10.1093/schbul/13.3.383 3629195

[52] Bauer DJ, Curran PJ. The integration of continuous and discrete latent variable models: Potential problems and promising opportunities. Psychological Methods. 2004;9: 3–29. 10.1037/1082-989X.9.1.3 15053717

[53] McLaughlin KA, Sheridan MA. Beyond cumulative risk: A dimensional approach to childhood adversity. Current Directions in Psychological Science. 2016;25: 239–245. 10.1177/0963721416655883 27773969 PMC5070918

[54] McLaughlin KA, Sheridan MA, Lambert HK. Childhood adversity and neural development: deprivation and threat as distinct dimensions of early experience. Neuroscience & Biobehavioral Reviews. 2014;47: 578–591. 10.1016/j.neubiorev.2014.10.012 25454359 PMC4308474

